# Robust Adaptive Cubature Kalman Filter and Its Application to Ultra-Tightly Coupled SINS/GPS Navigation System

**DOI:** 10.3390/s18072352

**Published:** 2018-07-20

**Authors:** Xin Zhao, Jianli Li, Xunliang Yan, Shaowen Ji

**Affiliations:** 1 School of Nuclear Engineering, Rocket Force University of Engineering, Xi’an 710025, China; zhaoxin20062111@163.com; 2 School of Instrument Science and Opto-electc Engineering, Beihang University, Beijing 100191, China; jishaowen@buaa.edu.cn; 3 School of Astronautics, Northwestern Polytechnical University, Xi’an 710072, China; xly_nwpu@126.com

**Keywords:** cubature Kalman filter, adaptive filter, hypersonic, ultra-tightly coupled, integrate navigation

## Abstract

In this paper, we propose a robust adaptive cubature Kalman filter (CKF) to deal with the problem of an inaccurately known system model and noise statistics. In order to overcome the kinematic model error, we introduce an adaptive factor to adjust the covariance matrix of state prediction, and process the influence introduced by dynamic disturbance error. Aiming at overcoming the abnormality error, we propose the robust estimation theory to adjust the CKF algorithm online. The proposed adaptive CKF can detect the degree of gross error and subsequently process it, so the influence produced by the abnormality error can be solved. The paper also studies a typical application system for the proposed method, which is the ultra-tightly coupled navigation system of a hypersonic vehicle. Highly dynamical scene experimental results show that the proposed method can effectively process errors aroused by the abnormality data and inaccurate model, and has better tracking performance than UKF and CKF tracking methods. Simultaneously, the proposed method is superior to the tracing method based on a single-modulating loop in the tracking performance. Thus, the stable and high-precision tracking for GPS satellite signals are preferably achieved and the applicability of the system is promoted under the circumstance of high dynamics and weak signals. The effectiveness of the proposed method is verified by a highly dynamical scene experiment.

## 1. Introduction

The state estimation of nonlinear systems is widely used in engineering applications, such as radar target tracking, complex image processing, highly dynamical navigation and positioning, and signal processing. In order to obtain the optimal estimation of a nonlinear system, the posterior probability distribution of the system state needs to be obtained. However, a complete description of the posterior probability distribution can be accurately known only in a few specific cases. In recent years, driven by engineering applications, a large number of suboptimal filtering methods have been proposed, which can be classified into two main categories: The first one is the linearization method such as the extended Kalman filter (EKF). The second one is the sampling method, i.e., the unscented Kalman filter (UKF) and particle filter (PF). EKF is a traditional approach to solving nonlinear problems, such as navigation, target tracking, information fusion, monitoring state estimation [1,2], etc. However, in a system with a higher degree of nonlinearity, the EKF will have a larger truncation error under a low carrier to noise ratio, which can easily cause the filter to diverge. In addition, the EKF implementation needs to calculate the Jacobian matrix, which also limits its scope of applications. The second method directly uses nonlinear filtering algorithms to process observation information, which can improve the accuracy of state estimation. Among them, UKF approximates the distribution function of random variables through a set of determined weighted sampling points [3]. When the sampling points are propagated through a nonlinear function, the statistical properties of the non-linear functions are captured, and the precision of UKF can reach the third-order [4,5]. However, UKF still has some limitations. In high-dimensional systems (usually higher than three-dimensions), in order to avoid the propagation of non-positive definite covariance matrices, it should choose the parameters of UKF very carefully [6]. At this time, the UKF is prone to numerical instability, which can lead to dimensional disasters or divergence, and limited their application in complex systems, such as high dynamics, weak signals, and strong non-linearity. The PF is proposed based on the idea of the Monte Carlo method [7,8], which uses a large number of randomly-generated particles to approximate the posterior probability density. The PF is used to solve signal processing problems in nonlinear and non-Gaussian systems. In recent years, it has been widely used for target tracking, state estimation under low-dynamical conditions, and modality detection [9]. However, as the number of iterations increases, there will be particle degradation and depletion when using PF [10]. In order to solve the problem of particle degradation, many improved particle algorithms have emerged. However, the generation of a large number of particles will still increase the computational burden, and the reduction of degradation and depletion is at the cost of increasing the complexity of the PF algorithm. Therefore, it is difficult for PF to meet real-time requirements, especially for state estimation under highly dynamical conditions.

In [11], the authors proposed a new non-linear filtering method based on the cubature transformation: the cubature Kalman filter (CKF). Once proposed, the method was used for navigation [12,13,14], attitude estimation [15], continuous system [16], hybrid filtering [17], and so on. The CKF is similar to the UKF filtering process, but its theoretical derivation is more rigorous [18]. It generates new points by converting 2n equal-valued cubature points through nonlinear functions and is used to predict the system state at the next moment. The CKF also does not need to linearize the non-linear dynamics and has all the features of the UKF. CKF uses a symmetrical sampling strategy and has fewer sampling points than the UKF does. Compared with filtering algorithms, such as EKF, UKF, and PF, CKF has better nonlinear performance, higher numerical accuracy, and better filter stability, and it is relatively simple to implement.

However, when CKF is applied to a nonlinear system, it needs to know the mathematical model of the object to be studied and the prior knowledge of noise statistical properties. In practice, it is difficult to obtain the prior knowledge of the noises statistics. The inaccurate mathematical model and inaccurate noise statistics may lead to large state estimation error, or even divergence. To solve these problems, scholars further study the CKF technique combined with an adaptive filter. The typical research results are mainly divided into two types. The first types of typical results proposed are improved CKF algorithms based on the strong tracking filter (STF), which can be used to solve the filter divergence problem caused by inaccurate system models. Aiming at the problem of CKF precision degradation caused by model uncertainty, the literature [19] established a strong tracking CKF algorithm (STCKF). The literature [20] proposed a strong tracking adaptive CKF algorithm viewing STF as the basic theory framework and making D replace EKF, and the algorithm improved the filtering performance of the existing system model uncertainty. To overcome the outliers caused by the model uncertainties, the literature [21] designed a robust strong tracking CKF and developed a noise statistic estimator based on the principle of maximum a posterior. The CKF used in the above method are all three-degree algorithms. Further, to improve the estimation accuracy, Cui and Zhang [22] proposed an improved high-degree CKF combined with the STF algorithm, named as the adaptive high-degree CKF (AH-CKF). In the AH-CKF, by introducing the STF into the high-degree CKF and modifying the predicted states’ error covariance with a fading factor, the residual sequence is forced to be orthogonal so that the robustness of the filter and the capability to deal with uncertainty factors are improved. However, these results are only for research on low- and medium-dynamic applications.

The second types of typical research results are improved CKF methods based on the robust M-estimation technique. Huang [23] proposed a class of robust CKF algorithms with statistical regression to solve the problem that the conventional CKF declines in accuracy and further diverges when the noise is not Gaussian noise. Wu [24] proposed a robust CKF based on generalized M-estimation to reduce the influence of measurement outliers on a target tracking system. Similarly, in order to address the degradation of the standard CKF due to outliers in measurement, Li [25] presented a robust version of CKF using Huber’s M-estimation methodology and square-root filtering framework. Essentially, the above research results are all effective improvements to the standard CKF or square-root CKF using he robust M-estimation technique. The solved problem is the degradation or divergence of the standard CKF caused by the inaccuracy of measurement noise. However, these robust M-estimation methods cannot adjust the noise covariance adaptively when it does not match with the truth. The problem will become more severe in the case of significant outliers [26]. To overcome this deficiency, Zhang and Zhi [26] came up with a new way and proposed an adaptive Huber’s M-estimation-based CKF (AHCKF) which can automatically adjust the measurement noise and gain adaptivity. The AHCKF has enhanced outlier robustness, reliability, and high estimate accuracy. This method, however, does not consider the problem of when the model uncertainties exist.

The above two types of improved CKF methods are developed respectively for the uncertainty of the model and the inaccuracy of measurement noise. However, these two kinds of problems often exist in the actual system synchronously, such as a highly dynamical navigation system or high maneuvering target tracking system, typically. To overcome this deficiency, we propose a novel, robust, adaptive CKF compromising the robust M estimation and the adaptive adjustment factor. The contribution of the paper can be summarized as follows:

1. The proposed algorithm could solve the problems of the model uncertainty and the measurement noise statistics inaccuracy. To handle abnormal measurement noise, we adopt robust M-estimation to automatically adjust the measurement noise covariance and gaining adaptivity. Compared with the literature [23,24,25,26], we directly use innovation to calculate the equivalent weight matrix, no-demand partial derivative operation, and iterative operation. To overcome model uncertainty, we derive an adaptive adjustment factor to modify the model as a whole. Compared with STF in the literature [19,20,21,22], the derived adaptive adjustment factor is simpler.
2. The proposed algorithm has lower computational complexity, and so it is suitable for dynamic systems with high real-time requirements. Our algorithm is not simply a cumulative combination of existing methods, but a derivation method considering the mathematical complexity and real-time, which ensures the computational efficiency of the algorithm.
3. In the application of the algorithm, we designed a stable and reliable ultra-tightly coupled structure based on a double-modulating loop. The effectiveness of the proposed method and the designed structure is verified by a highly dynamical scene experiment.

The rest of the paper is organized as follows: Section 2 gives a brief introduction of the Bayesian filter theory in the Gaussian domain and the cubature Kalman filter. Then, the improved robust adaptive CKF, using robust estimation theory and adaptive filter techniques, is also derived in Section 3. In Section 4, the hypersonic vehicle state estimation problem is studied using a SINS-aided GPS ultra-tightly coupled system, and a SINS/GPS federated ultra-tightly coupled structure is designed based on a double-modulating loop. In Section 4, we also give the nonlinear pre-filter tracking loop model of the satellite signal. In Section 5, the proposed robust adaptive CKF is applied in the highly dynamical nonlinear state estimation experiment. The conclusion of the paper is given in Section 6.

## 2. CKF Algorithm

### 2.1. Bayesian Estimation Rule

In this paper, we consider the nonlinear dynamic system, whose state space model is defined by the difference equation in discrete time [27]:

(1) $$\begin{array}{l} x_{k}=f(x_{k-1},u_{k-1})+v_{k-1}\\ z_{k}=h(x_{k},u_{k})+w_{k} \end{array}$$

where $x_{k}$ and $z_{k}$ are the n-dimensional state and the m-dimensional measurement of the dynamic system, respectively; $u_{k}$ is the n-dimensional control input; $\left\{v_{k-1}\right\}$ is a n-dimensional system noise sequence with zero mean and covariance $Q_{k-1}$; $\left\{w_{k}\right\}$ is an m-dimensional measurement noise sequence with zero mean and covariance $R_{k}$; f(•) and h(•) are the nonlinear state function and measurement function of the system, respectively.

When $v_{k-1}$ and $w_{k}$ are independent, the posterior density of the state $x_{k}$ of the nonlinear dynamic model (1) can be obtained based on the Bayesian estimation rule as follows:

(1) *Time update*, calculating the predictive density:
  (2) $$p(x_{k}|D_{k-1})=\int p(x_{k},x_{k-1}|D_{k-1})dx_{k-1}=\int p(x_{k-1}|D_{k-1})\times p(x_{k}|x_{k-1},u_{k-1}),dx_{k-1}$$
  where $D_{k-1}={\left\{u_{i},z_{i}\right\}}_{i=1}^{\left(k-1\right)}$ describes the history data up to time (k−1); $p(x_{k-1}|D_{k-1})$ is the old posterior density at time (k−1); the state transition density $p(x_{k}|x_{k-1},u_{k-1})$ is calculated by Equation (1).
(2) Measurement update
  The posterior density of the current state can be obtained by Bayesian rule:
  (3) $$p(x_{k}|D_{k})=\frac{1}{c_{k}}p(x_{k}|D_{k-1},u_{k-1})p(z_{k}|x_{k},u_{k})$$
  $$c_{k}=\int p(x_{k}|D_{k-1},u_{k})p(z_{k}|x_{k},u_{k})dx_{k}$$
  where $c_{k}$ is a constant. However, for nonlinear/non-Gaussian cases, the computational complexity of $c_{k}$ is typically prohibitive. A computationally feasible approximation is provided by PF [28]. Since the denominator is a normalizing constant, Equation (3) can be regarded as an unnormalized density, the fact does not affect the following derivation.

If the $p(x_{k}|D_{k})$ can be calculated exactly, we obtain the state estimation and covariance matrix of $x_{k}$ as follows:

(4) $${\hat{x}}_{k}=\int x_{k}p(x_{k}|D_{k})dx_{k}$$

(5) $$P_{k+1}=\int \left(x_{k}-{\hat{x}}_{k}\right){\left(x_{k}-{\hat{x}}_{k}\right)}^{T}p(x_{k}|D_{k})dx_{k}$$

In fact, the analytic solutions for $p(x_{k}|D_{k})$ is difficult to be obtained in the nonlinear system. Therefore, we need to approximate the state estimation and covariance matrix.

### 2.2. CKF Algorithm Based on a Third-Order Spherical-Radial Cubature Rule

Here, we consider the Gaussian noise, and it is widely used in Bayesian filter theory. The key approximation is $p(x_{k}|D_{k-1})$ and $p(z_{k}|D_{k})$ are both Gaussian in the Bayesian filter, which deduces a Gaussian $p(x_{k}|D_{k})$.

Suppose an n-dimensional random vector $x_{k}$ obeys the Gaussian distribution $N(x_{k};{\hat{x}}_{k},P_{x})$, we can obtain:

(6) $${\hat{x}}_{k|k-1}=E\left[f(x_{k-1},u_{k-1})|D_{k-1}\right]=\int f(x_{k-1},u_{k-1})p(x_{k-1}|D_{k-1})dx_{k-1}=\int f(x_{k-1},u_{k-1})N(x_{k-1};{\hat{x}}_{k-1|k-1},P_{k-1|k-1})dx_{k-1}$$

The error covariance is:

(7) $$\begin{array}{l} P_{k|k-1}=E\left[(x_{k}-{\hat{x}}_{k|k-1}){\left(x_{k}-{\hat{x}}_{k|k-1}\right)}^{T}|z_{1:k-1}\right]\\ =\int f(x_{k-1},u_{k-1})f^{T}(x_{k-1},u_{k-1})\times N(x_{k-1};{\hat{x}}_{k-1|k-1},P_{k-1|k-1})dx_{k-1}-{\hat{x}}_{k|k-1}{\hat{x}}^{T}{}_{k|k-1}+Q_{k-1} \end{array}$$

The predicted measurement can be obtained as follows:

(8) $${\hat{z}}_{k|k-1}=\int h(x_{k},u_{k})N(x_{k};{\hat{x}}_{k|k-1},P_{k|k-1})dx_{k-1}$$

The associated covariance and the cross-covariance are:

(9) $$P_{zz,k|k-1}=\int h(x_{k},u_{k})h^{T}(x_{k},u_{k})\times N(x_{k};{\hat{x}}_{k|k-1},P_{k|k-1})dx_{k}-{\hat{z}}_{k|k-1}{\hat{z}}^{T}{}_{k|k-1}+R_{k-1}$$

(10) $$P_{xz,k|k-1}=\int x_{k}h^{T}(x_{k},u_{k})\times N(x_{k};{\hat{x}}_{k|k-1},P_{k|k-1})dx_{k}-{\hat{x}}_{k|k-1}{\hat{z}}_{k|k-1}^{T}$$

We can see that Equations (6) to (10) have the unified integral form as:

(11) $$I(g)=\int g(x)N(x;\hat{x},P_{x})dx$$

It is difficult to obtain the analytic of the above multivariate integral, and the approximate calculation is needed to be adopted.

The CKF algorithm is derived from the idea of approximate calculation. CKF uses the third-order spherical-radial cubature rule to approximately compute the posterior mean and the covariance matrix for the nonlinear Gaussian system. The key step is to select a series of cubature points. Then we substitute these cubature points into a nonlinear function, calculate, and obtain some new cubature points which are used to approximately compute the integral values through the weighting method. The main steps of CKF are summarized as follows.

The CKF algorithm includes two processes: the time update and the measurement update [29]. The time updating is as follows:

(1) The posteriori probability distribution of a given *k* − 1 moment is assumed to be:
  $$p(x_{k-1}|{\left\{u_{i},z_{i}\right\}}_{i=1}^{k-1})=ℑ(x_{k-1};{\hat{x}}_{k-1|k-1},P_{k-1|k-1})$$
  Let:
  (12) $$P_{k-1|k-1}=S_{k-1|k-1}S_{k-1|k-1}^{T}$$
(2) Calculating state volume points:
  (13) $$X_{j,k-1|k-1}^{\hbar }=S_{k-1|k-1}\xi_{j}+{\hat{x}}_{k-1|k-1}$$
(3) Volume points transformed based on the state equation:
  (14) $$X_{j,k-1|k-1}^{*}=f(X_{j,k-1|k-1}^{\hbar },u_{k-1})$$
(4) Weighted mean to compute the state quantity prediction value:
  (15) $${\hat{x}}_{k|k-1}=\frac{1}{2n}\sum_{j=1}^{2n}X_{j,k-1|k-1}^{*}$$
(5) Calculate the covariance matrix of state prediction:
  (16) $$P_{k|k-1}=\frac{1}{2n}\sum_{j=1}^{2n}X_{j,k-1|k-1}^{*}X_{j,k-1|k-1}^{*T}-{\hat{x}}_{k|k-1}{\hat{x}}^{T}{}_{k|k-1}+Q_{k-1}$$

**Measurement updating:**

(1) Matrix factorization:
  (17) $$P_{k|k-1}=S_{k|k-1}S_{k|k-1}^{T}$$
(2) Calculate volume points:
  (18) $$X_{j,k|k-1}^{\hbar }=S_{k|k-1}\xi_{j}+{\hat{x}}_{k|k-1}$$
(3) Transform volume points based on the measurement equation:
  (19) $$Z_{j,k-1|k-1}^{*}=h(X_{j,k|k-1})$$
(4) Forecast the measurement value:
  (20) $${\hat{z}}_{k|k-1}=\frac{1}{2n}\sum_{j=1}^{2n}Z_{j,k|k-1}^{*}$$
(5) Calculate the prediction residual covariance matrix:
  (21) $$P_{zz,k|k-1}=\sum_{j=1}^{2n}w_{j}Z_{j,k|k-1}^{*}Z_{j,k|k-1}^{*T}-{\hat{z}}_{k|k-1}{\hat{z}}_{k|k-1}^{T}+R_{k}$$
(6) Calculate the cross covariance matrix:
  (22) $$P_{xz,k|k-1}=\sum_{j=1}^{2n}w_{j}X_{j,k|k-1}^{\hbar }Z_{j,k|k-1}^{*T}-{\hat{x}}_{k|k-1}{\hat{z}}_{k|k-1}^{T}$$
(7) Calculate the Kalman filter gain:
  (23) $$W_{k}=P_{xz,k|k-1}P_{zz,k|k-1}^{-1}$$
(8) Update the state:
  (24) $${\hat{x}}_{k|k}={\hat{x}}_{k|k-1}+W_{k}(z_{k}-{\hat{z}}_{k|k-1})$$
(9) Update the state covariance matrix:
  (25) $$P_{k|k}=P_{k|k-1}-W_{k}P_{zz,k|k-1}W_{k}^{T}$$

## 3. Novel Robust Adaptive CKF

When CKF is applied to a nonlinear system, it needs to know the mathematical model of the object to be studied and the prior knowledge of the noise statistics. However, if the filter is solved based on an inaccurate mathematical model and inaccurate noise statistics, it may result in a large state estimation error or even divergence. To solve this problem, it is necessary to study the adaptive filtering. For typical dynamic application systems, we focus on the study of adaptive filtering technology under the influence of two types of errors, that is, measuring abnormal errors and dynamic model errors.

It is inevitable to produce the gross error in the observations for a dynamic system. For example, when tracking a satellite signal, it is shown that the occurrence of gross errors accounts for 1% to 10% of the total number of observations [30]. To a certain extent, the uncertainty factors are introduced into the statistical characteristics of the measurement noise. Therefore, it should be adjusted on line adaptively. In addition, in the condition of high dynamics and low SNR, the satellite signal tracking of the observed information is very easy to be influenced by a poor environment, and there is a large proportion of noise, so that the noise statistics deviate from the prior statistical characteristics. In serious cases, an abnormal perturbation error may exist. The above problem limits the application of CKF, so it is necessary to adjust the measurement noise in real-time to enhance the ability of the algorithm to resist gross and abnormal errors.

In view of the gross error, robust M estimation in robust estimation theory is applied to the CKF algorithm, which can detect the influence degree of gross error, and then the model is adaptively adjusted and corrected, so as to eliminate the influence of the abnormal observation error on the algorithm. The improved CKF algorithm is called the robust CKF algorithm. In what follows, we will derive the algorithm. Since the measurement information only affects the updating process of the measurement, compared with the standard CKF algorithm, the robust CKF algorithm only adjusts and modifies the relevant expressions in the measurement updating equation as follows:

(26) $${\bar{P}}_{zz,k|k-1}=\sum_{j=1}^{2n}w_{j}Z_{j,k|k-1}^{*}Z_{j,k|k-1}^{*T}-{\hat{z}}_{k|k-1}{\hat{z}}_{k|k-1}^{T}+{\bar{R}}_{k}$$

where ${\bar{R}}_{k}$ is an equivalent measurement noise variance matrix corresponding to $R_{k}$, and it can be obtained by obtaining an equivalent weight matrix $\bar{P}$ in an anti-difference M estimation method. That is:

(27) $${\bar{R}}_{k}={\bar{P}}^{-1}$$

For the calculation of the equivalent weight matrix, the common methods are the IGGIII method, Andrew method, Tukey method, and Huber method [31]. Considering that the first three methods can make the diagonal elements of the p matrix 0, the Huber method can guarantee that the diagonal elements of $\bar{P}$ matrices are positive. The expression of equivalent is as follows:

(28) $${\bar{p}}_{t_{ii}}=\{\begin{array}{c} \begin{array}{cc} \begin{array}{c} \begin{array}{cc} 1/\sigma_{ii} & \end{array} \end{array} & \left|r_{i}/\sigma_{r_{i}}\right|=\left|r_{i}^{\prime }\right|\leq c \end{array}\\ \begin{array}{cc} \begin{array}{c} \begin{array}{cc} c/\sigma_{ii}\left|r_{i}^{\prime }\right| & \end{array} \end{array} & \left|r_{i}^{\prime }\right|>c \end{array} \end{array}$$

(29) $${\bar{p}}_{t_{ij}}=\{\begin{array}{c} \begin{array}{cc} \begin{array}{c} \begin{array}{cc} 1/\sigma_{ij} & \end{array} \end{array} & \left|r_{i}^{\prime }\right|\leq c\;\mathrm{and}\;\left|r_{j}^{\prime }\right|\leq c \end{array}\\ \frac{c}{\sigma_{ij}\max\left\{\left|r_{i}^{\prime }\right|,\left|r_{j}^{\prime }\right|\right\}}\begin{array}{cc} & \left|r_{i}^{\prime }\right|>c\;\mathrm{or}\;\left|r_{j}^{\prime }\right|>c \end{array} \end{array}$$

where ${\bar{p}}_{t_{ii}}$ and ${\bar{p}}_{t_{ij}}$ are diagonal elements and non-diagonal elements of $\bar{P}$, respectively; $\sigma_{ii}$ and $\sigma_{ij}$ are diagonal elements and non-diagonal elements of the original $R_{k}$ array; $r_{i}$ is a residual component corresponding to the observation quantity $Z_{i}$, $r_{i}^{\prime }$ is a corresponding standard residual component, and $\sigma_{r_{i}}$ is a mean square error of $r_{i}$; and *c* is a given constant, usually taken from 1.3 to 2.0.

The above operation involves that $\sigma_{r_{i}}$ and $r_{i}$ are deterministic. In practice, because the covariance matrix of the measurement residuals is obtained from Equation (21), that is, the variable quantity $P_{zz,k|k-1}$ before being modified, the expressions $\sigma_{r_{i}}$ and $r_{i}$ are:

(30) $$\sigma_{r_{i}}={\left(P_{zz,k|k-1}\right)}_{ii}$$

(31) $$r_{i}={\left(z_{k}-{\hat{z}}_{k|k-1}\right)}_{i}$$

Then, ${\bar{P}}_{zz,k|k-1}$ is substituted for $P_{zz,k|k-1}$ in Equation (15), and the gain matrix is modified. Furthermore, the subsequent filtering solution is continued. The robust CKF algorithm is actually based on the standard CKF filter and modifies the noise covariance matrix $R_{k}$, so as to adjust the filter gain matrix, and ultimately enhance the performance of algorithm against observation errors consisting in satellite signal to restrain the divergence of Kalman filter. Therefore, robust CKF algorithm is more suitable for the near space environment, in which satellite signals are unstable and measurement noise statistical characteristics deviate considerably from the prior information.

The robust CKF algorithm solves the influence of the observation abnormal errors in the system. In other words, aiming at the inaccurate measurement noise, the CKF algorithm was improved, and then completing the improvement of the CKF algorithm when there are errors in the dynamic model. It is notable that the inaccuracy of system noise statistical characteristics is also an inducement of dynamic model errors.

The dynamic equations established for the satellite signal link may deviate greatly from the real model of the system when a nearby space vehicle makes a highly dynamic maneuvering flight, that is to say, a large dynamic disturbance error may exist in the state equation of the system, and the CKF filter algorithm fixed by the model cannot estimate the state parameters of the system. Therefore, based on the above robust CKF algorithm, a robust adaptive CKF algorithm is proposed for dynamic model errors.

It is pointed out in [13] that the error of the dynamic model usually destroys the effect of parameter estimation as a whole; in other words, the errors of the dynamic model will affect the estimation of all state parameter components. Therefore, we consider using an adaptive adjustment factor to modify the model as a whole, which also ensures the computational efficiency of the algorithm. The specific algorithm is that adaptive regulator $\vartheta_{k}$ modify $P_{k|k-1}$, obtaining:

(32) $${\overset{⌢}{P}}_{k|k-1}=\vartheta_{k}^{-1}P_{k|k-1}=\vartheta_{k}^{-1}\left[\frac{1}{2n}\sum_{j=1}^{2n}X_{j,k-1|k-1}^{*}X_{j,k-1|k-1}^{*T}-{\hat{x}}_{k|k-1}{\hat{x}}_{k|k-1}^{T}+Q_{k-1}\right]\;$$

where the optimal value of $\vartheta_{k}$ may be obtained from the residual covariance matrix after predicting and updating, where $\vartheta_{k}$ is given by the following theorem.

**Theorem** **1.** *If* ${\hat{P}}_{zz,k|k-1}$ *is the residual covariance matrix estimated after the introduction of new measurement information, it is defined as the updating residual covariance matrix;* ${\tilde{P}}_{zz,k|k-1}$ *is a theoretical residual covariance matrix obtained by adaptive filtering, and* $P_{zz,k|k-1}$ *is a residual covariance matrix obtained by the covariance propagation law. The selection of the optimal adaptive factor shall ensure that the following equation holds:*

(33) $${\tilde{P}}_{zz,k|k-1}={\hat{P}}_{zz,k|k-1}$$

*Then, the optimal adaptive factor is:*

(34) $$\vartheta_{k}=\frac{tr(P_{zz,k|k-1}-{\bar{R}}_{k})}{tr({\hat{P}}_{zz,k|k-1}-{\bar{R}}_{k})}$$

**Proof** **of** **Theorem** **1.** Let ${\tilde{x}}_{k|k-1}$ a step-by-step prediction error, then:

(35) $${\hat{x}}_{k|k-1}=x_{k}-{\tilde{x}}_{k|k-1}$$

Thus, the filtered residual is:

(36) $$r_{k}=z_{k}-h({\hat{x}}_{k|k-1})=z_{k}-h(x_{k}-{\tilde{x}}_{k|k-1})$$

A first-order Taylor expansion of the second term on the right-hand side of the equation:

(37) $$h(x_{k}-{\tilde{x}}_{k|k-1})\approx h(x_{k})-\frac{\partial h}{\partial x}|{}_{x={\hat{x}}_{k|k-1}}\times {\tilde{x}}_{k|k-1}=h(x_{k})-D_{k}\times {\tilde{x}}_{k|k-1}$$

Bringing the above equation into the following equation:

(38) $$r_{k}=z_{k}-h(x_{k})+D_{k}{\tilde{x}}_{k|k-1}=v_{k}+D_{k}{\tilde{x}}_{k|k-1}$$

Then the residual covariance matrix A is:

(39) $$P_{zz,k|k-1}=E\left\{r_{k}r_{k}^{T}\right\}=E\left\{\left(v_{k}+D_{k}{\tilde{x}}_{k|k-1}\right){\left(v_{k}+D_{k}{\tilde{x}}_{k|k-1}\right)}^{T}\right\}=E\left(D_{k}{\tilde{x}}_{k|k-1}{\tilde{x}}_{k|k-1}^{T}D_{k}^{T}\right)+E\left(v_{k}v_{k}^{T}\right)=D_{k}P_{k|k-1}D_{k}^{T}+{\bar{R}}_{k}$$

Note the equation using the measurement noise covariance matrix after being modified by robust CKF. Furthermore, in adaptive filtering, $P_{k|k-1}$ is modified to $\vartheta_{k}^{-1}P_{k|k-1}$, and then the theoretical residual covariance matrix obtained by adaptive filtering is:

(40) $${\tilde{P}}_{zz,k|k-1}=\vartheta_{k}^{-1}D_{k}P_{k|k-1}D_{k}^{T}+R_{k}$$

From the equation:

(41) $${\hat{P}}_{zz,k|k-1}={\tilde{P}}_{zz,k|k-1}=\vartheta_{k}^{-1}D_{k}P_{k|k-1}D_{k}^{T}+R_{k}$$

Multiply the two sides of the upper form by A and move the item to obtain

(42) $$\vartheta_{k}({\hat{P}}_{zz,k|k-1}-R_{k})=D_{k}P_{k|k-1}D_{k}^{T}$$

Further derivation:

(43) $$\vartheta_{k}({\hat{P}}_{zz,k|k-1}-R_{k})=D_{k}P_{k|k-1}D_{k}^{T}=P_{zz,k|k-1}-{\bar{R}}_{k}$$

Take the traces of the matrices on both sides of the expressions. Then, the expression of the optimal adaptive factor can be obtained by the migration transformation. ☐

In practical application, the regulatory factor in adaptive filtering algorithm is usually not greater than 1 [32,33], so we further determine the optimal adaptive factor as follows:

(44) $$\vartheta_{k}=\{\begin{array}{c} 1\begin{array}{cc} & tr({\hat{P}}_{zz,k|k-1})\leq tr(P_{zz,k|k-1}) \end{array}\\ \frac{tr(P_{zz,k|k-1}-{\bar{R}}_{k})}{tr({\hat{P}}_{zz,k|k-1}-{\bar{R}}_{k})}\begin{array}{cc} & tr({\hat{P}}_{zz,k|k-1})>tr(P_{zz,k|k-1}) \end{array} \end{array}$$

Taking into account the fact that the numerator and the denominator in the upper equation all contain the variance term of measurement noises. The approximate optimal adaptive factor expression can also be obtained by using the common polynomial.

(45) $$\vartheta_{k}\approx \{\begin{array}{c} 1\begin{array}{cc} & tr({\hat{P}}_{zz,k|k-1})\leq tr(P_{zz,k|k-1}) \end{array}\\ \frac{tr(P_{zz,k|k-1})}{tr({\hat{P}}_{zz,k|k-1})}\begin{array}{cc} & tr({\hat{P}}_{zz,k|k-1})>tr(P_{zz,k|k-1}) \end{array} \end{array}$$

${\hat{P}}_{zz,k|k-1}$ can be obtained by the residual error estimation of the observations. The sliding window method is adopted, namely:

(46) $$tr({\hat{P}}_{zz,k|k-1})=tr(r_{k}r_{k}^{T})=r_{k}^{T}r_{k}=\sum_{i=1}^{m}{\left(z_{k}-{\hat{z}}_{k|k-1}\right)}_{i}$$

In order to further clarify the robust adaptive CKF algorithm, the following steps are given:

**Step** **1.** Set the initial condition.
  (47) $${\hat{x}}_{0}=E(x_{0})P_{0}=E{\left[\left(x_{0}-{\hat{x}}_{0})(x_{0}-{\hat{x}}_{0}\right)\right]}^{T}$$
**Step** **2.** Forecast the update.
  For a given A and B, according to Equations (12)–(26), the state prediction value C and its prediction covariance matrix D are obtained.
**Step** **3.** Calculate the state volume points in the measurement updating.
  The matrix decomposition of the state prediction covariance matrix is completed by using Equation (17), and replacing Equation (18) with the following equation, adaptive adjustment of the model will be complete:
  (48) $$X_{j,k|k-1}^{\hbar }=\sqrt{\vartheta_{k}^{-1}}S_{k|k-1}\xi_{j}+{\hat{x}}_{k|k-1}$$
**Step** **4.** Calculate the predicted values and the residual covariance matrix.
  Computational observational prediction ${\hat{z}}_{k|k-1}$ and predictive residual covariance matrix $P_{zz,k|k-1}$ are calculated by Equations (19)~(21).
**Step** **5.** Robust correction.
  The robust correction of the algorithm is completed according to the Equations (26)–(29).
**Step** **6.** Adaptive factor regulation model.
  Use the approximate expression Equation (45) to calculate the optimal adaptive factor, if the value is 1, then continue the following steps; if the value is less than 1, the value is substituted into Equation (48), and then by the Equations (19), (20) and (26) calculate the adaptive factor to adjust the revised prediction residual covariance matrix.
**Step** **7.** Update the measurement.
  According to Equations (22)–(25), the update calculation of the state variable and its corresponding state covariance matrix is completed.

**Remark** **1.** *The proposed robust adaptive cubature Kalman filter (CKF) could deal with the problem of inaccurately known system model and noises statistics simultaneously. The proposed method includes two steps of improvement for the basic CKF. First, in order to overcome the kinematic model error, we introduce an adaptive factor to adjust the covariance matrix of state prediction, and process the influence introduced by the dynamic disturbance error. Second, aiming at overcoming the abnormality error, we adopt the robust estimation theory to adjust the CKF algorithm online. Our algorithm is not simply a cumulative combination of existing methods, but a derivation method considering the mathematical complexity and real-time, which ensures the computational efficiency of the algorithm and is more suitable for highly dynamical application scenarios.*

## 4. SINS/GPS Ultra-Tightly Coupling Structure Based on a Double Loop

### 4.1. Design of SINS/GPS Ultra-Tightly Coupling Structure Based on a Double Loop

In this section, we present a typical application of the proposed method, that is, the application to an ultra-tightly coupled SINS/GPS navigation system of a hypersonic vehicle.

In the literature [34], the federated ultra-tightly coupled structure is divided into three types. In essence, three kinds of structures are implemented only in signal tracking error estimation, which use three different ways to estimate code and carrier correlation error. Other parts are essentially identical. The realization of satellite signal tracking uses a closed loop mode of an external loop, which is a typical vector tracking structure, and this kind of structure enhances the mutual assistance of satellite signal tracking between channels. However, at the same time, there is a problem that when the number of unstable channels is large, if the carrier to noise ratio of multiple satellite signals is reduced due to strong interference, the unstable channel dominates in the signal tracking process. The accuracy of the integrated navigation filter will be reduced, and using this single information to adjust the local signal generator of all channels, the tracking performance of a few normal channels will be affected, and the performance of integrated navigation system will further deteriorate. Therefore, the design of this kind of single regulating loop has a certain “inverse” effect on the tracking process of the satellite signal under special circumstances. In addition, the GPS tracking loop of this kind of structure is an open loop [35,36]. If the integrated navigation filter, for some reason, such as failure, cannot work normally, the entire system will be paralyzed, and the reliability of the system will reduce.

**Figure 1:**
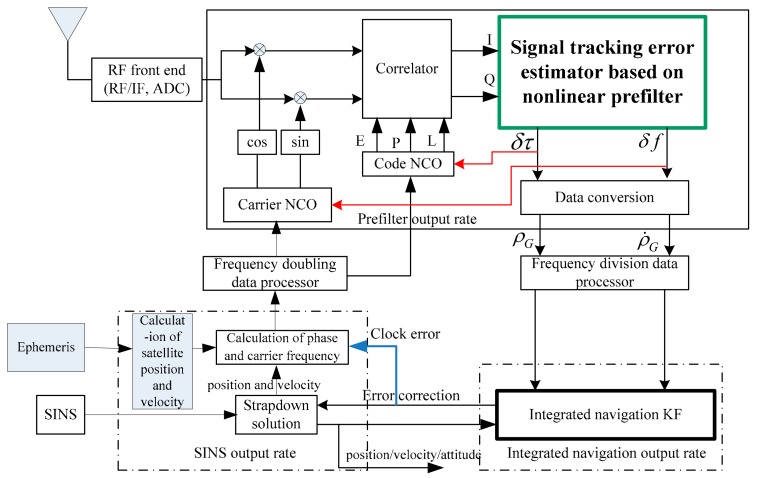
SINS/GPS federated ultra-tightly coupled structure based on double loop.

In order to reduce the influence of the “inverse” action mentioned above, we take the advantages of the conventional scalar tracking structure and design the ultra-tightly coupled structure based on the double loop adjustment method shown in Figure 1. One path is aimed at the internal loop of each channel, which is like the channel tracking of the conventional receiver. We use the method of mutual auxiliary tracking between the code and carrier closed loop, and the tracking error that is estimated by the pre-filter of each channel to adjust the code/carrier NCO in real-time, in which the pre-filter adopts a non-linear filtering model. The other path is the external loop described above, which uses the position and velocity information output by the corrected SINS and per the ephemeris information to update and correct, so as to adjust the code phase and the carrier frequency information of the NCO. The structure design of the double loop determines the code/carrier NCO adjustment in two ways, one of which achieves a single internal loop adjusting mode through the pre-filter, and the other way is a combined adjustment mode of external loop supporting internal loop with SINS participating. The strategy of adjustment is to read the corrected inertial navigation information in the program design. If the information meets the accuracy level requirement of NCO adjustment, the combination adjustment method will be adopted. If not, it will depend on the internal loop to adjust. This strategy can reduce the influence of the “inverse” function existing in the common vector tracking method and improve the reliability of the integrated navigation system.

### 4.2. Pre-Filter Tracking Modeling

The above pointed out that the pre-filter tracking algorithm is one of the key points of ultra-tightly coupled systems. Thus, this section gives the nonlinear pre-filter tracking loop model of the satellite signal.

(1) Pre-filter state equation establishment
  State variables take:
  (49) $$X={\left[\begin{array}{ccccc} A & \delta \tau & \delta \phi & \delta f & \delta a \end{array}\right]}^{T}$$
  where A denotes the normalized amplitude of the received signal, the code phase error; δf the carrier wave frequency error, and δa the carrier phase acceleration error. The equation of state of the system is:
  (50) $$\begin{array}{ll} \dot{X} & ={\left[\begin{array}{ccccc} \dot{A} & \delta \dot{\tau } & \delta {\dot{\phi }}_{0} & \delta {\dot{f}}_{0} & \delta {\dot{a}}_{0} \end{array}\right]}^{T}=FX+Gw\\ & =\left(\begin{array}{ccccc} 0 & 0 & 0 & 0 & 0\\ 0 & 0 & 0 & \beta & 0\\ 0 & 0 & 0 & 1 & 0\\ 0 & 0 & 0 & 0 & 1\\ 0 & 0 & 0 & 0 & 0 \end{array}\right)\left(\begin{array}{c} A\\ \delta \tau \\ \delta \phi_{0}\\ \delta f_{0}\\ \delta a_{0} \end{array}\right)+\left(\begin{array}{ccccc} 1 & 0 & 0 & 0 & 0\\ 0 & 1 & \beta & 0 & 0\\ 0 & 0 & 1 & 0 & 0\\ 0 & 0 & 0 & 1 & 0\\ 0 & 0 & 0 & 0 & 1 \end{array}\right)\left(\begin{array}{c} w_{A}\\ w_{\tau }\\ w_{\phi_{0}}\\ w_{f_{0}}\\ w_{a_{0}} \end{array}\right) \end{array}$$
  where β is the transformation coefficient that converts the radian to the chip, and the components of w are the amount of the process noise corresponding to the state variables.
(2) Pre-filter measurement equation establishment
  The system’s observation is obtained by in-phase signal *I* and quadrature signal *Q* outputting by the correlator. *I* and *Q* include the early (*E*) signal, the present (*P*) signal and the later (*L*) signal. The expression of the measurement equation is as follows:
  (51) $$\left[\begin{array}{l} I_{P}\\ I_{E}\\ I_{L}\\ Q_{P}\\ Q_{E}\\ Q_{L} \end{array}\right]=\left[\begin{array}{c} ATD(t_{k}-\tau )R(\delta \tau )\sin c(\delta fT/2)\cos(\delta fT/2+\delta \phi )\\ ATD(t_{k}-\tau )R(\delta \tau -0.5\Delta )\sin c(\delta fT/2)\cos(\delta fT/2+\delta \phi )\\ ATD(t_{k}-\tau )R(\delta \tau +0.5\Delta )\sin c(\delta fT/2)\cos(\delta fT/2+\delta \phi )\\ ATD(t_{k}-\tau )R(\delta \tau )\sin c(\delta fT/2)\sin(\delta fT/2+\delta \phi )\\ ATD(t_{k}-\tau )R(\delta \tau -0.5\Delta )\sin c(\delta fT/2)\sin(\delta fT/2+\delta \phi )\\ ATD(t_{k}-\tau )R(\delta \tau +0.5\Delta )\sin c(\delta fT/2)\sin(\delta fT/2+\delta \phi ) \end{array}\right]+\left[\begin{array}{c} n_{I_{P}}\\ n_{I_{E}}\\ n_{I_{L}}\\ n_{Q_{P}}\\ n_{Q_{E}}\\ n_{Q_{L}} \end{array}\right]$$
  where *T* is the cumulative sum of time; $D(t_{k}-\tau )$ is the navigation message modulation; Δ is the difference number of code between the early and the later codes, R(*) respectively correspond with the results of the received C/A code sequence and the locally-generated code in E code, P code, and L code after being relative to each other.

## 5. Experimental Analysis of a Highly Dynamical Scene

A near space vehicle often flies at high speed. This section of the experiment will focus on the carrier of high maneuverability factors on the impact of signal tracking, while considering that the lower the signal carrier noise ratio, the more difficult the stable tracking of the signal, and the more able to assess the performance of the research method. To this end, a lower signal to noise ratio is set to verify the tracking performance of the proposed robust adaptive CKF method under the condition of a highly dynamic, and low carrier, noise ratio.

### 5.1. Experimental Scheme

**Figure 2:**
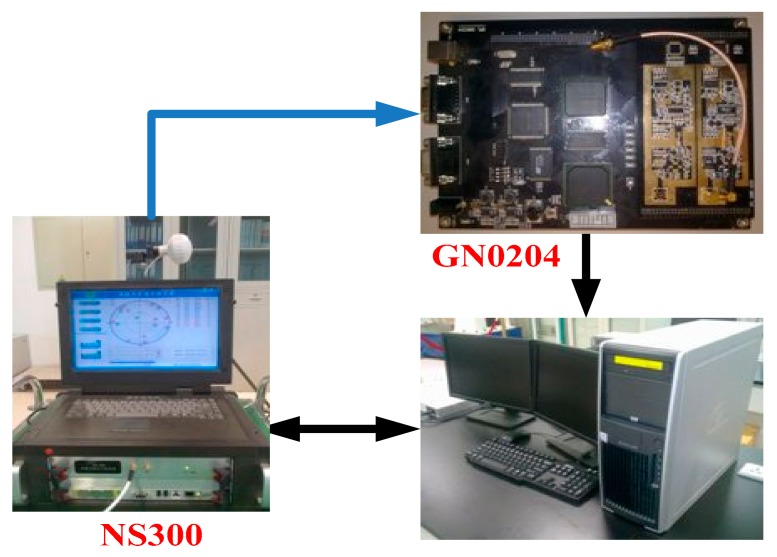
Structure of the highly-dynamical scene experiment scheme.

The NS300 multimode satellite signal simulator generates a highly dynamical GPS RF signal, and the RF signal is received by the GN0204 satellite signal receiving device, converted into an intermediate frequency signal and stored. Then we use the SINS/GPS ultra-tight coupling software to receive the intermediate frequency signal processed by the system platform so that it is convenient for further testing of the proposed coupling structure and the adaptive performance of the algorithm for highly dynamical conditions. In the experiment, the SINS information is generated by the digital emulator, and the information of SINS and GPS is generated according to the pre-established standard track, so as to ensure the information equivalence between them at the same time. The structure of the scheme is shown in Figure 2.

The signal tracking experiment is compared based on the generated data with five algorithm combinations: The first method is the designed ultra-tightly coupled structure based on the double loops, and the proposed robust adaptive CKF algorithm is used to estimate and track parameters, which is abbreviated as DLB-RACKF. The structures of the second method and third method are the same as the first method. The difference is that the algorithms used in the filtering algorithms are CKF and UKF, respectively, similarly, the corresponding methods are abbreviated to DLB-CKF and DLB-UKF. To prove the superiority of the proposed algorithm, we have further completed the comparison between similar algorithms. The fourth and fifth algorithms are respectively selected from two typical improved CKFs mentioned in the introduction, namely the STCKF algorithm proposed by [19] and the AHCKF algorithm proposed by [26]. Similarly, the corresponding methods are abbreviated to DLB-STCKF and DLB-AHCKF.

### 5.2. Setup of the Experimental Conditions

Major parameter settings of flight path: the initial position is 102.0266 °E, 28.2460 °N, height: 50 km, and the initial velocity (in the geographical coordinate system) is 0 m/s in the east, 0 m/s in the north, and 0 m/s in the sky.

Select 60 s flight data of variable acceleration motion. The specific geographical coordinates are set as follows: the acceleration in the north direction and in the sky direction is constant between 0 s to 60 s, which is AN = 4 m/s^2^ in the north direction acceleration and AU = 0 m/s^2^ in the day direction acceleration, respectively. Eastward is variable acceleration motion, in which 0–20 s, 21–40 s, and 41–60 s are all constant acceleration motion, and eastward accelerations are AE = 100 m/s^2^, AE = 200 m/s^2^, and AE = −200 m/s^2^, respectively. 20–21 s and 40–41 s are the constant jerk motions, the numerical value of which are 100 m/s^3^ and −400 m/s^3^, respectively. As a result, the maximum absolute speed is about 6000 m/s, the maximum absolute acceleration is about 20 *g*, and the maximum jerk is about 40 *g*/s, which is fully in line with the highly dynamical characteristics.

The satellite signal simulator is used to generate the satellite signal which can be received by the highly dynamic moving carrier at the current time, and the carrier to noise ratio (C/N_0_) of all the satellite signals is set to 20 dB-Hz. There are currently 13 visible satellites in the sky. In addition, in order to verify the proposed method of adaptive adjusting performance, the measurement noise parameters are expanded by 20 times among 5–10 s, so that gross error of observations are simulated equivalently; on the other hand, the high maneuvering scenario set in this section can evaluate the adaptive performance of the algorithm when the dynamic model is imprecise, so it is not necessary to set the dynamic model error.

### 5.3. Experimental Results and Analysis

Since the performance of each tracking channel is the same and the satellite signal index is consistent, the results of one of the satellite channels is selected here in order to analyze the results.

**Figure 3:**
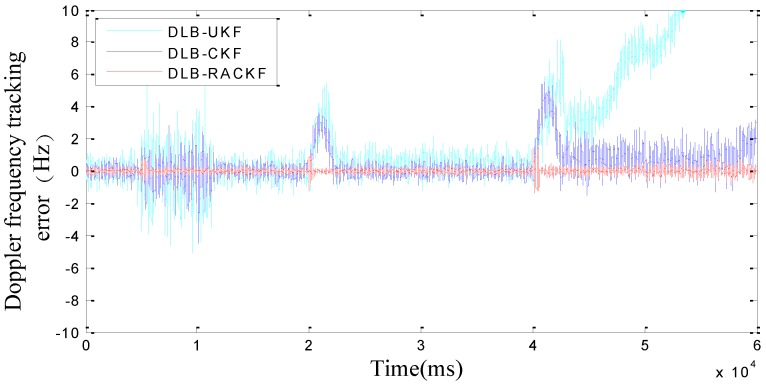
Comparison of the Doppler frequency tracking error among DLB-UKF, DLB-CKF, and DLB-RACKF.

**Figure 4:**
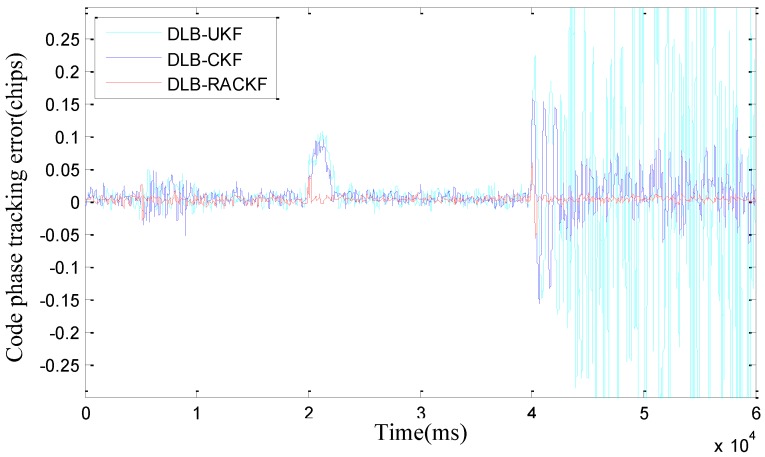
Comparison of the code phase tracking error among DLB-UKF, DLB-CKF, and DLB-RACKF.

**Figure 5:**
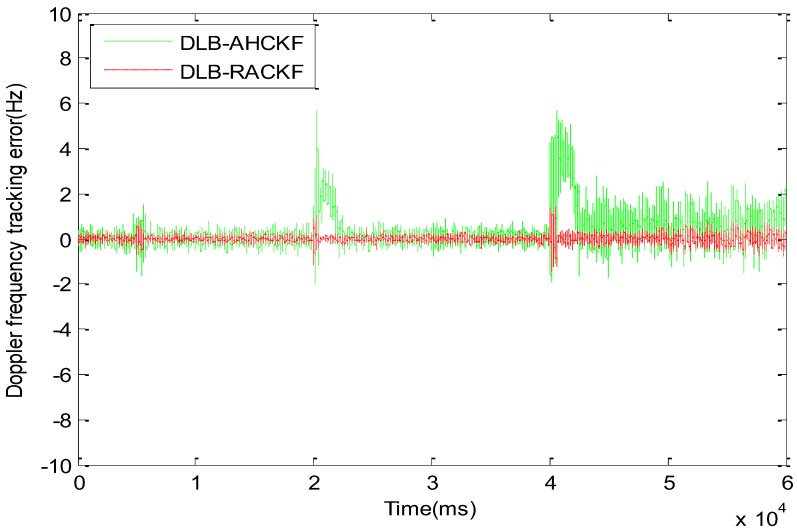
Comparison of code phase tracking error among DLB-AHCKF and DLB-RACKF.

**Figure 6:**
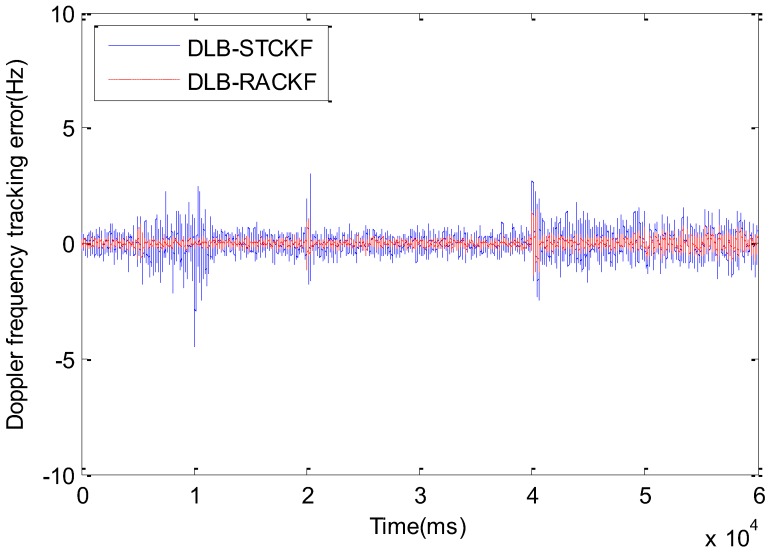
Comparison of code phase tracking error among DLB-STCKF and DLB-RACKF.

The comparison results of DLB-RACKF, DLB-CKF, and DLB-UKF tracking errors are shown in Figure 3 and Figure 4. Figure 5 and Figure 6 show the comparison results of DLB-RACKF, DLB-AHCKF, and DLB-STCKF. Table 1 shows the RMS statistics of the tracking error of the five methods in each period. The runtime performance of five filters is summarized in Table 2.

Figure 3 and Figure 4 are essentially based on the comparison results of tracking errors among three different algorithms with the same coupling structure. From Figure 3 and Figure 4 and Table 1, we can see that the accuracy of DLB-CKF is slightly better than that of DLB-UKF in tracking error accuracy. The precision of DLB-RACKF is better than that of DLB-CKF and DLB-UKF. In the tracking stability of the algorithm, due to the lack of adaptive adjustment performance, both DLB-CKF and DLB-UKF show the condition of changing errors when the measurement noise increases and the vehicle moves with high maneuverability, such as when the 5th second measurement noise parameter continues to expand, the phase frequency and code tracking error of DLB-CKF and DLB-UKF immediately change. Eventually it will lead to a decline in the precision of navigation. When the noise of the 10th second measurement returns to normal, the PB-KF-UTC does not return to the initial accuracy immediately, but after about 1000 ms, it gradually converges to the normal precision range. In the 20th second, when the carrier performs variable acceleration motion in the range of about 10 g/s, the tracking error of PB-KF-UTC suddenly increases. This is due to the fact that the dynamic model no longer meets the basic situation of the actual motion, there is a large deviation, and when the variable acceleration motion is finished, the error of DLB-CKF and DLB-UKF is converged to the normal precision range again, with the process lasting for 2 s to 3 s. In the same way, in the 40th second, when the carrier performs variable acceleration motion at the range of about 10 g/s, the tracking error of DLB-CKF and DLB-UKF changes again, but because the maneuverability is too large, with the result that it is difficult for PB-KF-UTC to converge again when the motion of variable acceleration is finished, the phenomenon of divergence appears. However, in the whole tracking process, DLB-RACKF has maintained a good tracking accuracy and stability. The DLB-CKF converges gradually after the end of the variable acceleration motion, which ensures continuous tracking, which indicates that the stability of CKF is better than that of UKF. Compared with DLB-CKF and DLB-UKF, DLB-RACKF is relatively stable in the whole tracking process. When the measurement noise is abnormal and the dynamic model error increases due to the high maneuvering motion of the carrier, the DLB-RACKF can maintain better adaptive regulation performance. Its tracking stability is much better than that of DLB-CKF and DLB-UKF.

Figure 5 and Figure 6 compare the tracking errors among the improved CKF algorithms with the same adaptive adjustment function. As shown in Figure 5 and Table 1, DLB-AHCKF and DLB-RACKF exhibit good adaptive performance when the kinematic model is accurate and the measurement error increases. Since the 5th second of measurement noise continues to increase, the DLB-AHCKF accurately estimates the noise changes after about 800 ms, and adjusts the algorithm to the normal range of precision. In the 20th and 40th seconds, when the carrier is highly maneuvering for two times, the dynamic model is no longer accurate, and the tracking error of DLB-AHCKF increases suddenly. The change rule is similar to that of DLB-CKF. After the first maneuver, the tracking error of the DLB-AHCKF gradually converges to the normal range. However, the tracking error of the second maneuver fails to converge normally, and the trend of the divergence of the algorithm appears. The result of this experiment is determined by the fundamental principles of the DLB-AHCKF algorithm, and it has its own specific application background. On the contrary, as shown in Figure 6 and Table 1, the DLB-CKF algorithm is essentially proposed for the motion state mutation of the carrier or the inaccurate model. Thus, when the highly dynamical variable acceleration takes place twice, the DLB-STCKF shows good tracking performance similar to that of the DLB-RACKF. However, when the system model is accurate and the measurement noise exists, the tracking error of DLB-STCKF is always greater than the normal mean for the duration of abnormal noise, which is similar to DLB-RACKF. When the noise returns to normal, there is a convergence delay of about 1300 ms. In general, compared with DLB-AHCKF and DLB-STCKF, the DLB-RACKF algorithm can deal with abnormal measurement noise and inaccurate motion models simultaneously. Within the normal range, the precision of the three algorithms is roughly equivalent, and the DLB-RACKF is slightly better than the other two algorithms.

As shown in Table 2, the running time of the CKF algorithm is lower than that of the UKF under the same software operation conditions. The running time of the improved RACKF algorithm is about 1/2 of UKF. The computational efficiency of DLB-RACKF is better than that of DLB-AHCKF and DLB-STCKF, which is especially important for state estimation under highly dynamical conditions. Therefore, RACKF is an effective nonlinear state estimator.

## 6. Conclusions

The design of an ultra-tight coupling structure realized by a single regulating loop has a certain “inverse” effect on tracking satellite signals under special circumstances. In this paper, a cascade super-tight coupling structure based on double loop adjustment was designed, which reduces the “inverse” effect of the common vector tracking method and improves the reliability of the integrated navigation system.

At the same time, in order to improve tracking accuracy, a new nonlinear filtering algorithm called CKF was studied. The robust M estimation in robust estimation theory is used to improve the CKF, which solves the influence of the abnormal error of the observed quantity. On the other hand, an adaptive adjustment factor was used to modify the algorithm, which can effectively deal with the influence of the dynamic disturbance error. The simulation results of highly dynamical scenes based on the satellite signal simulator show that the proposed structure and the proposed robust adaptive CKF algorithm effectively improve the tracking accuracy and tracking stability of the GPS satellite signal so that the navigation system has better dynamic adaptability.

## Figures and Tables

**Table 1:** Satellite Doppler frequency shift and code phase tracking error RMS.

Satellite Signal Tracking Error Terms Corresponded to Four Methods		0–20 s	20–40 s	40–60 s
Doppler shift errors (Hz)	DLB-UKF	0.9153	1.0574	
	DLB-CKF	0.5345	0.7375	1.5053
	DLB-AHCKF	0.2483	0.6547	1.3267
	DLB-STCKF	0.4755	0.3003	0.5004
	DLB-RACKF	0.1125	0.1220	0.2455
Code phase errors (chips)	DLB-UKF	0.0128	0.0277	0.2413
	DLB-CKF	0.0123	0.0245	0.0474
	DLB-RACKF	0.0091	0.0060	0.0077

**Table 2:** Runtime of five tracking algorithms.

Algorithms	DLB-UKF	DLB-CKF	DLB-AHCKF	DLB-STCKF	DLB-RACKF
**Runtime (s)**	1.832600	0.7332	1.2109	1.2973	0.9368
